# Supplementary material for: Assessing workflow impact and clinical utility of AI-assisted brain aneurysm detection: A multi-reader study
Source: Neuroimage Clin. 2025 Jun 28;47:103835. doi: 10.1016/j.nicl.2025.103835 (PMC12271490; doi:10.1016/j.nicl.2025.103835)
Supplement: Supplementary Data 1 [file mmc1.docx]

**Supplementary Materials**

**Table A:** MR acquisition parameters of TOF-MRA scans of our study sample (training, validation and test sets).

| **Dataset** | **# scans** | **Vendor** | **Model** | **Field strength [T]** | **Median**  **TR [ms]** | **Median**  **TE [ms]** | **Voxel spacing**  **[**$\boldsymbol{mm}^{\boldsymbol{3}}$**]** |
| --- | --- | --- | --- | --- | --- | --- | --- |
| **Training Dataset** | 69 | Philips | Intera | 3.0 | 379 | 3.0 | 0.29 x 0.29 x 4.0 |
|  | 61 | Siemens  Healthineers | Verio | 3.0 | 400 | 3.0 | 0.56 x 0.56 x 3.9 |
|  | 46 | Siemens  Healthineers | Skyra | 3.0 | 400 | 2.0 | 0.78 x 0.78 x 3.9 |
|  | 35 | Siemens  Healthineers | TrioTim | 3.0 | 440 | 3.0 | 0.56 x 0.56 x 3.9 |
|  | 32 | Siemens  Healthineers | Symphony | 1.5 | 400 | 14.0 | 0.45 x 0.45 x 6.0 |
|  | 24 | Siemens  Healthineers | Aera | 1.5 | 477 | 8.0 | 0.78 x 0.78 x 3.3 |
|  | 12 | Siemens Healthineers | Prisma | 3.0 | 400 | 2.0 | 0.78 x 0.78 x 3.3 |
| **Validation Dataset** | 41 | Siemens  Healthineers | Skyra | 3.0 | 400 | 2.0 | 0.78 x 0.78 x 3.9 |
|  | 22 | Siemens  Healthineers | Verio | 3.0 | 400 | 3.0 | 0.56 x 0.56 x 3.9 |
|  | 13 | Siemens Healthineers | Prisma | 3.0 | 400 | 2.0 | 0.78 x 0.78 x 3.3 |
|  | 5 | Siemens  Healthineers | Aera | 1.5 | 477 | 8.0 | 0.78 x 0.78 x 3.3 |
| **Test Dataset** | 53 | Siemens  Healthineers | Skyra | 3.0 | 400 | 2.0 | 0.78 x 0.78 x 3.9 |
|  | 24 | Siemens Healthineers | Prisma | 3.0 | 400 | 2.0 | 0.78 x 0.78 x 3.3 |
|  | 17 | Siemens  Healthineers | Verio | 3.0 | 400 | 3.0 | 0.56 x 0.56 x 3.9 |
|  | 6 | Siemens  Healthineers | Aera | 1.5 | 477 | 8.0 | 0.78 x 0.78 x 3.3 |

**Table B**: complete list of locations provided to the readers. This was used to indicate the location(s) of the spotted aneurysm(s). For instance, if an aneurysm was found in the right branch of the Middle Cerebral Artery (MCA), the reader would report it with the number 16.

| **Location** | **Code** |
| --- | --- |
| NO aneurysm found | 0 |
| Extradural Carotid left | 1 |
| Extradural Carotid right | 2 |
| Intradural Carotid other  (carotid cave, hypophysaire sup, chroroidal) left | 3 |
| Intradural Carotid other  (carotid cave, hypophysaire sup, chroroidal) right | 4 |
| Ophthalmic Carotid left | 5 |
| Ophthalmic Carotid right | 6 |
| Carotid tip left | 7 |
| Carotid tip right | 8 |
| PCOM left | 9 |
| PCOM right | 10 |
| ACOM | 11 |
| Pericallosal | 12 |
| ACA other left | 13 |
| ACA other right | 14 |
| MCA + MCA other left | 15 |
| MCA + MCA other right | 16 |
| PICA left | 17 |
| PICA right | 18 |
| post other (SCA, AICA, basilar trunk, PCA) left | 19 |
| post other (SCA, AICA, basilar trunk, PCA) right | 20 |
| Basilar tip | 21 |

**Section A – Transfer Learning Experiments**

Here, we describe the four transfer learning experiments that were carried out:

- **Mixed training:** the DL model was trained only once on a mixed dataset composed of the source ADAM dataset (N=113) and our training dataset (N=279), as previously done in [1].
- **Finetuning encoder:** the DL model was first fully trained on the source ADAM dataset (N=113), and then only the encoder of our 3D-UNET was finetuned on the in-house training dataset (N=279), while the decoder was frozen (i.e. not trained again). A similar approach was found to be beneficial in [2].
- **Finetuning decoder:** the DL model was first fully trained on the source ADAM dataset (N=113), and then only the decoder was finetuned on the in-house training dataset (N=279), while the encoder was frozen. This approach proved effective in [3].
- **Finetuning all layers:** the DL model was first trained on the source ADAM dataset (N=113), and then all its layers were finetuned on the in-house training dataset (N=279). This approach was also adopted by [4].

**Section B – Creation of overlay sequence**

The predicted aneurysm candidates were shown in the overlay sequence as red contours and, for each candidate, an associated probability. The contours were directly obtained from the segmentation mask with MeVisLab. Instead, the probability value is computed as the mean of the original probabilistic prediction (for each candidate), which in turn is computed as the mean prediction coming from overlapping patches during the sliding-window approach performed at inference time. On top of this, every predicted patch is previously computed as the mean volume after test-time augmentation; namely, every predicted patch is an average across:

- Model prediction on original patch
- Model prediction on horizontally flipped patch
- Model prediction on vertically flipped patch
- Model prediction on 90° rotated patch
- Model prediction on 180° rotated patch
- Model prediction on 270° rotated patch
- Model prediction on contrast-adjusted patch
- Model prediction on gamma-corrected patch
- Model prediction on patch where gaussian noise was added

**References**

[1] T. Di Noto *et al.*, “Weakly Supervised Learning with Automated Labels from Radiology Reports for Glioma Change Detection,” *arXiv*, 2023.

[2] B. Shirokikh, I. Zakazov, A. Chernyavskiy, I. Fedulova, and M. Belyaev, “First U-Net Layers Contain More Domain Specific Information Than The Last Ones,” *DART Work. - MICCAI*, Aug. 2020, [Online]. Available: http://arxiv.org/abs/2008.07357.

[3] K. Kushibar *et al.*, “Supervised Domain Adaptation for Automatic Sub-cortical Brain Structure Segmentation with Minimal User Interaction,” *Sci. Rep.*, vol. 9, no. 1, Dec. 2019, doi: 10.1038/s41598-019-43299-z.

[4] A. Abbas, M. M. Abdelsamea, and M. M. Gaber, “DeTrac: Transfer Learning of Class Decomposed Medical Images in Convolutional Neural Networks,” *IEEE Access*, vol. 8, pp. 74901–74913, 2020, doi: 10.1109/ACCESS.2020.2989273.
